# Propionate alleviates myocardial ischemia-reperfusion injury aggravated by Angiotensin II dependent on caveolin-1/ACE2 axis through GPR41

**DOI:** 10.7150/ijbs.67724

**Published:** 2022-01-01

**Authors:** Fan Deng, Liang-Qing Zhang, Han Wu, Yu Chen, Wen-Qian Yu, Rong-Hui Han, Yuan Han, Xiao-Qi Zhang, Qi-Shun Sun, Ze-Bin Lin, Yu Wang, Yong-Pan Liu, Jing-Yi Chen, Ke-Xuan Liu, Jing-Juan Hu

**Affiliations:** 1 Department of Anesthesiology, Nanfang Hospital, Southern Medical University, Guangzhou 510515, China.; 2 Department of Anesthesiology, Taihe Hospital, Hubei University of Medicine, Shiyan 442000, China.; 3 Department of Anesthesiology, Affiliated Hospital of Guangdong Medical University, Zhanjiang 524001, China.; 4 Department of Dermatology, Shunde Hospital, Southern Medical University, Foshan, China.; 5 The First Ward of Pain Department, Hubei NO. 3 People's Hospital of Jianghan University, Wuhan 430000, China.; 6 Department of Cardiology, State Key Laboratory of Organ Failure Research, Nanfang Hospital, Southern Medical University, Guangzhou 510515, China.; 7 Major of Clinical Medicine, Nanshan College, Guangzhou Medical University, Guangzhou 510515, China.

**Keywords:** Myocardial ischemia reperfusion, Angiotensin II, Caveolin-1, Angiotensin-converting enzyme 2, Propionate, G-protein coupled receptor 41.

## Abstract

Myocardial ischemia/reperfusion (I/R) injury is still a lack of effective therapeutic drugs, and its molecular mechanism is urgently needed. Studies have shown that the intestinal flora plays an important regulatory role in cardiovascular injury, but the specific mechanism has not been fully elucidated. In this study, we found that an increase in Ang II in plasma was accompanied by an increase in the levels of myocardial injury during myocardial reperfusion in patients with cardiopulmonary bypass. Furthermore, Ang II treatment enhanced mice myocardial I/R injury, which was reversed by caveolin-1 (CAV-1)-shRNA or strengthened by angiotensin-converting enzyme 2 (ACE2)-shRNA. The results showed that CAV-1 and ACE2 have protein interactions and inhibit each other's expression. In addition, propionate, a bacterial metabolite, inhibited the elevation of Ang II and myocardial injury, while GPR41-shRNA abolished the protective effects of propionate on myocardial I/R injury. Clinically, the propionate content in the patient's preoperative stool was related to Ang II levels and myocardial I/R injury levels during myocardial reperfusion. Taken together, propionate alleviates myocardial I/R injury aggravated by Ang II dependent on CAV-1/ACE2 axis through GPR41, which provides a new direction that diet to regulate the intestinal flora for treatment of myocardial I/R injury.

## Introduction

Cardiovascular disease has been the leading cause of human death worldwide, and treatment technologies for myocardial ischemia or infarction have been increasing over the past few decades 1, 2. Myocardial infarction treatment, cardiopulmonary bypass (CPB) and other diseases or treatment measures will inevitably lead to myocardial ischemia/reperfusion (I/R) 3-5. However, the cardiovascular reperfusion injury caused by excessive accumulation of oxygen free radicals and other factors is directly related to the patient's outcome, and it is one of the key stages to accelerate the recovery of patients. However, the mechanism and prevention strategies of myocardial I/R injury are not fully understood.

The mechanism of cardiovascular reperfusion injury is complex, but the currently accepted view is that oxygen free radical overload and inflammatory factor accumulation due to blood flow and oxygen recovery during reperfusion 6-8. Angiotensin II (Ang II), one of the most potent vasoconstrictors known, has been shown to be closely associated with oxidative stress and inflammation during reperfusion 9-11. The Ang II level in the myocardial I/R risk area is significantly higher than the non-ischemic left ventricle 12. Angiotensin-converting enzyme 2 (ACE2), which metabolizes and degrades Ang II, has been involved in myocardium I/R injury 13-15. However, changes in Ang II and ACE2 during myocardial I/R and their mechanisms of action on myocardial I/R injury have not yet been elucidated.

Studies have found that among the affected ACE2 network components related to the SARS-Cov-2 interaction group, angiotensinogen and caveolin-1 (CAV-1) are related to cardiovascular risk factors 16. Meanwhile, a novel ACE2 activator was confirmed to restore the expression of CAV-1 17. These suggests that the possible interaction between CAV-1 and ACE2 may be a potential regulatory mechanism for cardiovascular injury. However, protein interaction between ACE2 and CAV-1 during myocardial I/R injury has not been reported. CAV-1 is a marker protein of the caveolae of cardiovascular endothelial cells and is involved in many physiological and pathological processes, such as anti-fibrosis, inflammation and oxidative stress 3, 18. Furthermore, studies have confirmed that Ang II-infused wild-type mice have significant cardiovascular damage and inflammation, which were not found in CAV-1 knockout mice 19. Meanwhile, CAV-1 can competitively adsorb endothelial nitric oxide synthase (eNOS) and reduce the amount of NO, which can relax blood vessels and anti-inflammatory, in the blood 20. These indicate that CAV-1 may play a key role in Ang II-induced cardiovascular damage in myocardial I/R injury.

The gut microbiota has emerged as an important regulator of human physiology, and potentially deleterious alterations to the microbiota, commonly termed dysbiosis, are associated with numerous adverse physiological outcomes 21-25. More and more studies have shown that gut microbiota and its metabolites play an important role in myocardial I/R injury, and different diets may also affect the outcome of myocardial I/R injury 26-28. Short-chain fatty acids (SCFAs) are a major class of bacterial metabolites and are mainly produced in the colon by bacterial fermentation of otherwise indigestible polysaccharides (fibers) 29. The SCFA propionate (C3) is involved in the regulation of physiological or pathological processes such as cardiovascular inflammation, immune homeostasis, etc. 30-33. Furthermore, C3 can regulate the release of renin and the homeostasis of blood pressure.^34^, ^35^ It was recently identified the G-protein coupled receptor 41 (GPR41), which is the receptor for SCFAs, as a novel regulator of blood pressure.^36^, ^37^ However, it is unclear the role of C3 and its receptor GPR41 played in regulating Ang II levels and myocardial I/R injury. In summary, we aimed to observe the possibility of C3 and its receptor GPR41 in preventing myocardium I/R injury, and to explore its potential mechanism of affecting Ang II levels and regulating Ang II-induced myocardium I/R injury.

## Methods and Materials

### Approvals

All mice experimental procedures were performed in accordance with and approved by the Institutional Animal Care and Use Committee of Southern Medical University. All investigations involving the use of patient samples or tissues conformed to the principles outlined in the Declaration of Helsinki. The study protocol on clinical patient sample collection was approved by the Ethical Committee of Nanfang hospital, Southern Medical University (approval number NFEC-202009-k2-01). Written informed consent was obtained from all volunteers prior to the inclusion of subjects in the study.

### Patient samples

The study protocol was approved by the Ethical Committee of Nanfang hospital, Southern Medical University (approval number NFEC-202009-k2-01). The enrollment requirements of all patients who need cardiopulmonary bypass (CPB) surgery were carried out in accordance with the standards we established before 21, Participants were not included if they (1) <18 years old or >75 years old, (2) has chronic cardiovascular disease, previous heart surgery, and (3) used antidiarrheals, laxatives or prebiotics within 1 week, or used antibiotics within 3 months. For sample size of patients, we estimated that a total sample size ≥ 19 subjects would be required, assuming a correlation coefficient r=0.6 (α=0.05; 1-β=0.8). Finally, a total of 38 patients were enrolled. Collect the patient's stool on the day of surgery or the day before surgery, as well as blood samples during the perioperative period.

Collect blood samples from patients at 4 different time points: (T0) The patient has entered the operating room to adapt to the environment and is emotionally stable before anesthesia (5-10 minutes after entering the room); (T1) a stable state after the anesthesiologist performs general anesthesia and before the surgeon performs thoracotomy (5-10 minutes after general anesthesia); (T2) the CPB is performed and aorta is clamped for 30 minutes; (T3) the aorta is open for blood flow for 30 minutes. Detect the levels of CK-MB, cTnI and Ang II in patients' plasma.

### Establishment of myocardium I/R model

Healthy male C57BL/6 mice (weighing 20-25 g, 6-8 weeks old) were purchased from Southern Medical University and were housed in a temperature-controlled colony room on a 12/12-hour light-dark cycle. Mouse myocardium I/R model was performed as described.^38^ Briefly, the mice were anesthetized with 100% O_2_/4% isoflurane, and the whole process was maintained by administering 100% O_2_/2% isoflurane. A silk thread was used to ligate the left anterior descending coronary artery, 1 mm from the ascending aorta. Reperfusion for 2 hours after 30 minutes of ischemia. At the end of the protocol, all mice were euthanized by placing them under deep anesthesia with 100% O_2_/5% isoflurane.

At the beginning of myocardial reperfusion, mice were given 0.5 mg/kg of Ang II or solvent control to observe the effect of Ang II on myocardial I/R injury (Fig. S2A). In order to study the effects of C3, mice received sodium propionate (200 mmol/L, Sigma-Aldrich) or sodium chloride as a control (200 mmol/L, Sigma-Aldrich) in their drinking water ad libitum for 15 days (Fig. S3A) 39.

### Cardiac function assessment

Cardiac function assessment using a Vevo 2100 high-resolution imaging system equipped with a 30 MHz sensor with transthoracic echocardiography (RMV-707B; VisualSonics, Toronto, ON, Canada).

### Myocardial infarct size measurement

Myocardial infarct size was measured by 2, 3, 5-Triphenyl Tetrazolium Chloride (TTC) Staining 38. Specifically, the mouse heart was quickly cut off and evenly cut into 5 sections. The sections were gently rinsed with 0.9% saline and incubated in 2% TTC dye (Solarbio, Beijing, China) at 37 °C for 30 minutes in the dark. The non-infarcted area of myocardial tissue is stained red, while the infarcted area is gray-white. Use Image J software to measure the infarct area and total area of each piece of myocardial tissue, the infarct volume of each layer is the product of the infarct area and the thickness of the layer, the sum of the infarct volume of each layer is the total infarct volume. The infarct volume (%) = infarct volume/total volume.

### AAV9 and vector transfection

The recombinant adeno-associated virus serotype 9 (AAV9) vectors which carry a CMV promoter with GFP reporter (CAV-1-shRNA, ACE2-shRNA, GPR41-shRNA) (Hanbio Biotechnology Co., Shanghai, China) or GFP vector control (Hanbio Biotechnology Co.) to delete CAV-1 or ACE2 or GPR41 gene expression or as control.^38^ The mice were anesthetized with 2% isoflurane, and then the AAV9 vector (AAV9 negative control [shRNA-con] or AAV9-CAV-1 [CAV-1-shRNA, 5'-CCGCTTGTTGTCTACGATCTT-3'] or AAV9-ACE2 [ACE2-shRNA, 5'-GCCCAAAGTTTCTCACTACAA-3'] or AAV9-GPR41 [GPR41-shRNA, 5'-TTTGCTAAACCTGACCATTTC-3']) was randomly injected into the left ventricle of each animal at a virus dose of 1×10^11^ using an insulin syringe with a 30-gauge needle 3 weeks before the establishment of the myocardial I/R model. The AAV9 vector was injected into the heart at 5 locations, with a total injection volume of 20 μL per heart. After the operation, the skin was disinfected, and the animal was awakened by putting it on an insulation blanket.

### Detection of serum CK-MB, cTn-I, Ang II levels

The levels of CK-MB (CUSABIO BIOTECH CO.,Ltd, Wuhan, China), cTn-I (CUSABIO BIOTECH CO.,Ltd), and Ang II (CUSABIO BIOTECH CO.,Ltd) in the serum of patients and mice were performed according to the operating steps of the kit instructions.

### Detection of C3 content in the feces of patients

Detection of C3 content in feces were measured by gas chromatography/mass spectrometry (GC/MS) as described 21, 40. Briefly, 0.1 g feces were homogenized with 0.4 ml ddH2O and centrifuged at 4 °C and 5000 rpm for 5 minutes. Collect an aliquot (0.2 ml) of the supernatant, then add 0.05 ml 50% H2SO4 and 0.25 ml ether containing 50 μg/ml 2-methylvaleric acid. After vortexing for 2 minutes, the mixture was centrifuged at 4 °C and 12000 rpm for another 10 minutes. After incubating for 30 minutes at -20 °C, the supernatant was collected in a vial containing anhydrous sodium sulfate, and then applied to the Thermo TSQ Vantage triple quadrupole mass spectrometer. Data collection and processing were performed with TraceFinderTM software version 3.3 sp1 (Thermo Fisher Scientific Corp., USA). The C3 were quantified using pure standards diluted in ether.

### Superoxide dismutase (SOD) activity and malondialdehyde (MDA) activity

The SOD activity (Nanjing Jiancheng Bioengineering Institute, Nanjing, China) and MDA activity (Nanjing Jiancheng Bioengineering Institute) of the mouse myocardial tissue were tested according to the method of the kit instructions.

### Western blotting

The protein expression of CAV-1, ACE2 and GPR41 were detected by western blot. The RIPA lysis buffer (Solarbio) was used to extract total protein from cardiovascular tissue. The extracted protein was separated by 10% SDS-PAGE gel electrophoresis, then transferred to a PVDF membrane, blocked PVDM with 5% skimmed milk powder for 1 hour, and then incubated with CAV-1 (Sigma-Aldrich, Shanghai, China), ACE2 (Proteintech, Wuhan, China) and GPR41 (Abcam, Shanghai, China) antibodies at 4 ° C overnight. The next day, the PVDF membrane was cleaned three times with TBST (Solarbio) for 5 minutes each time. After incubating the correspondding secondary antibody at room temperature for 2 hours, the PVDF membrane was cleaned three times with TBST (Solarbio) for 5 minutes each time again. Observing protein bands using enhanced chemiluminescence (Thermo Fisher Scientific, Inc.).

### Immunohistochemistry

Immunohistochemistry were performed as previously described 21. Anti-CAV-1 antibody, anti-ACE2 antibody were used to detect protein expression in myocardial tissue. The Olympus microscope was used to capture images at 100 magnification, and 5 fields of view of each sample were randomly selected to quantify the relative intensity of protein staining.

### Co-immunoprecipitation

The co-immunoprecipitation experiment observed the interaction between ACE2 protein and CAV-1 protein in myocardial tissue was performed as described.^41^ Briefly, the myocardial tissue was lysed with RIPA buffer (Beyotime, Shanghai, China), and then centrifuged at 12,000×g for 15 minutes. Collect the supernatant and divide it into three aliquots for input, primary antibody, and control IgG. Incubate the primary antibody and IgG with protein A/G agarose beads on a shaker overnight at 4°C. After incubation, the sample was centrifuged at 3000×g for 5 min, the beads were collected, and washed 3 times with PBS. Add an appropriate amount of loading buffer to the beads, boil for 5 minutes, and then centrifuge at 12,000×g for 5 minutes to dissociate the protein from the beads. The IP product was then analyzed using Western blot.

### Statistical analysis

Data were analyzed and performed using GraphPad Prism software (version 7.0) by investigators blinded to the group allocation. The results are expressed as the mean ± SEM. Means of 2 continuous normally distributed variables were compared by independent samples Student's t-test. The Mann-Whitney U test and the Kruskal-Wallis test were used, respectively, to compare the means of 2 and ≤ 3 groups of variables that were not normally distributed. One-way Repeated Measures ANOVA was used to compare differences in myocardial injury and Ang II at different time points. In addition, the Spearman method was used for correlation statistical analysis. A value of *p* < 0.05 was considered significant.

## Results

### The dramatic increase level of Ang II is associated with aggravated myocardial injury during myocardial reperfusion in patients with CPB

**Figure 1:**
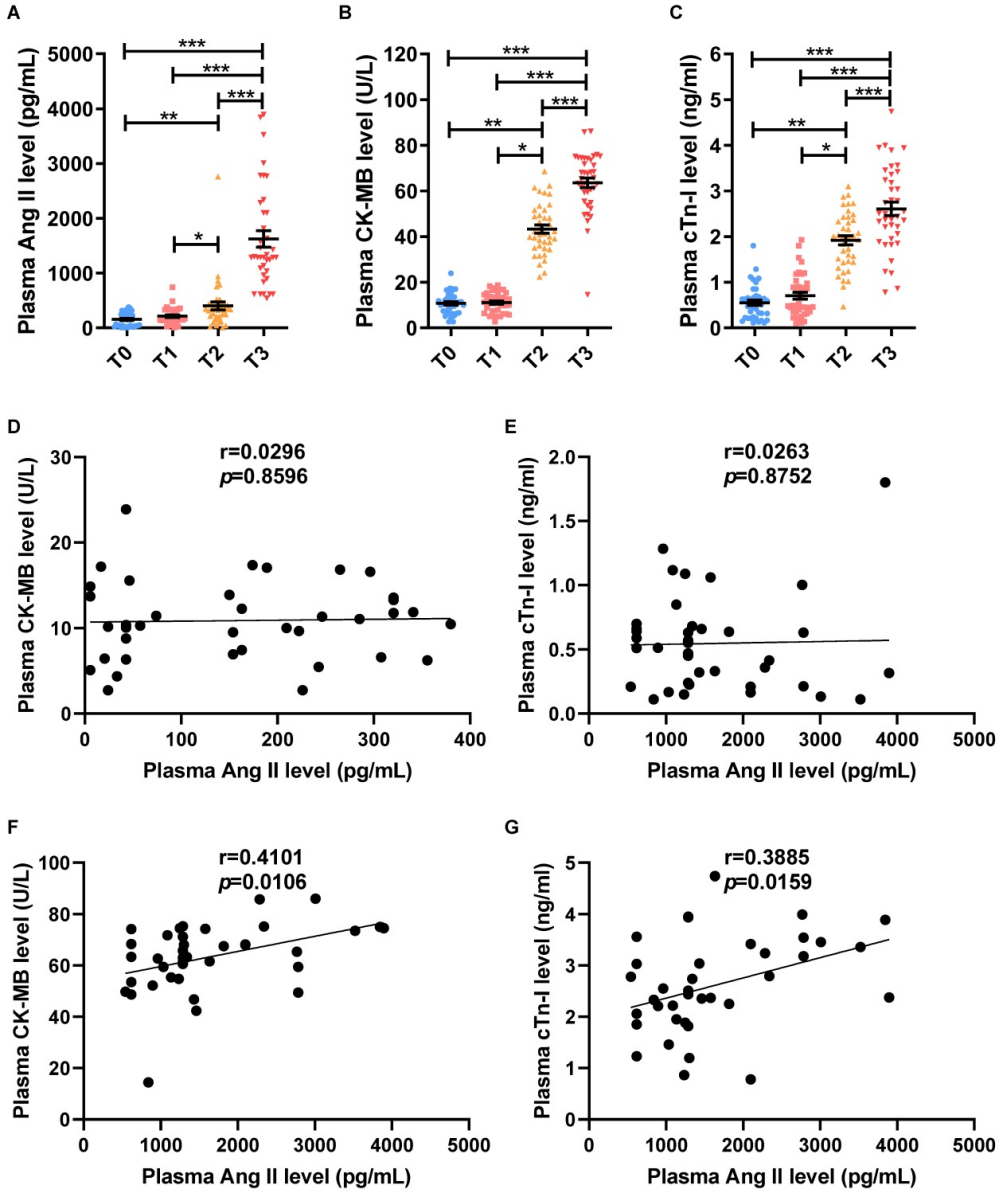
** The dramatic increase level of Ang II is associated with aggravated myocardial injury during myocardial reperfusion in patients with cardiopulmonary bypass.** (A) Ang II levels in the patient's plasma. (C-D) CK-MB levels and cTn-I levels in the patient's plasma. (D-E) The correlation analysis between the levels of Ang II and the levels of CK-MB (D), and cTn-I (E) in patient's plasma before surgery (T0). (F-G) The correlation analysis between the levels of Ang II and the levels of CK-MB (F), and cTn-I (G) in patient's plasma during myocardial reperfusion (T3). The results are expressed as the mean±SEM, n = 38. * *p* < 0.05, ** *p* < 0.01, *** *p* < 0.001 by spearman analysis.

To observe changes in Ang II levels and myocardial I/R in patients required valve replacement and CPB, a total of 38 patients who met the inclusion criteria were collected. Compared to T0, there was no significant difference in plasma Ang II level (Fig. 1A), plasma CK-MB level (Fig. 1B), plasma cTn-I level (Fig. 1C) and patient's heart rate (Fig. S1A) at T1, while systolic blood pressure, diastolic blood pressure and mean arterial pressure of patients were all significantly lower (Fig. S1B-D). Myocardial ischemia raised the level of Ang II in plasma (Fig. 1A), and increased the level of CK-MB and cTn-I in plasma at T2 (Fig. 1B-C), while the indicators of the heart could not be monitored due to cardiac ischemia arrest at T2. Compared to T0 or T1 or T2, the CK-MB level, cTn-I level and Ang II level in plasma were significantly increased at T3 (Fig. 1A-C). Furthermore, the patient's heart rate at T3 was higher, but systolic blood pressure, diastolic blood pressure, and mean arterial pressure were lower than T0 or T1 (Fig. S1A-D). In addition, there is no significant correlation between the Ang II level and CK-MB level (r=0.0296, *p*=0.8596), cTn-I level (r=0.0263, *p*=0.8752) in the patient's plasma before the operation at T0 (Fig. 1D-E). While the plasma Ang II levels in patients were positively correlated with plasma CK-MB level (r=0.4101, *p*=0.0106) and plasma cTn-I level (r=0.3885, *p*=0.0159) at T3 of myocardial reperfusion (Fig. 1F-G).

### Ang II perfusion aggravates myocardial I/R injury in mice

**Figure 2:**
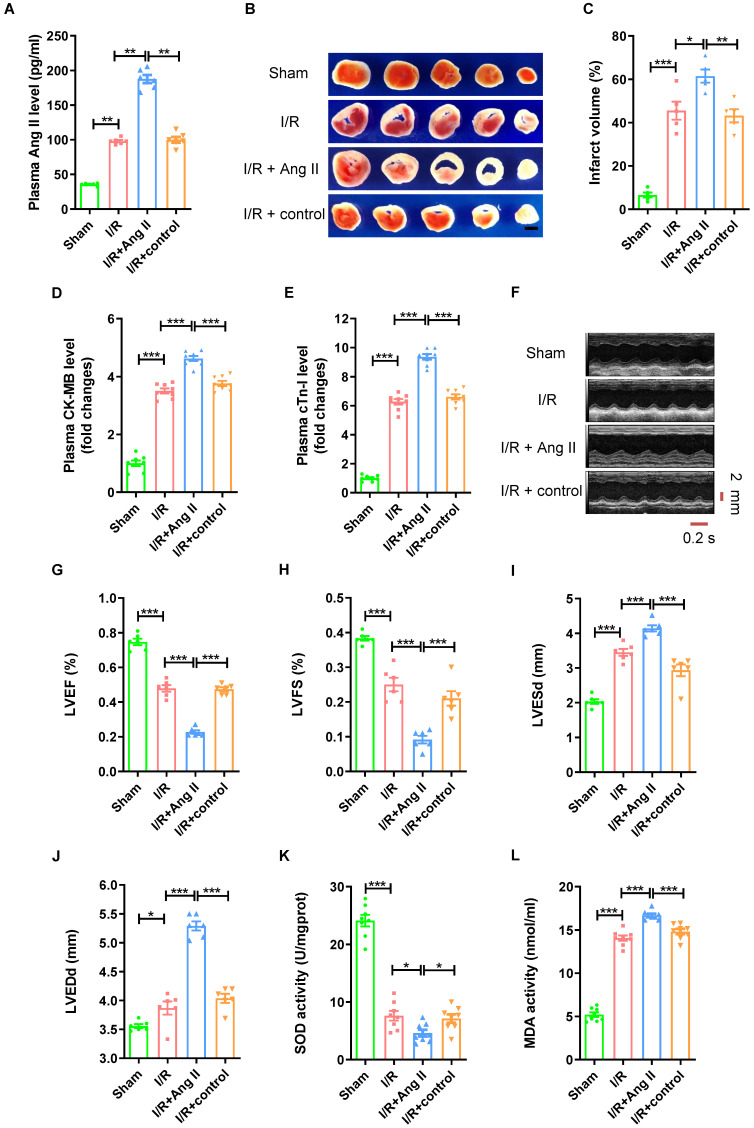
** Ang II perfusion aggravates myocardial I/R injury in mice.** (A) Ang II levels in mouse plasma. (B-C) TTC staining and myocardial infarction volume, scale bar is 5 mm, n = 5. (D-E) CK-MB levels and cTn-I levels in mouse plasma. (F-J) Cardiac function indicators left ventricular ejection fraction (LVEF), left ventricular fraction (LVFS), left ventricular end systolic diameter (LVESd) and left ventricular end diastolic diameter (LVEDd) were assessed by echocardiography, time stamp is 0.2 s and scale bar is 2 mm. (K-L) The superoxide dismutase (SOD) activity and malondialdehyde (MDA) activity of myocardial tissue. The results are expressed as the mean±SEM, n = 8. * *p* < 0.05, ** *p* < 0.01, *** *p* < 0.001 by one-way ANOVA (Tukey's test).

To further validate the role of Ang II in myocardial I/R injury, Ang II was injected intraperitoneally into mice at the beginning of myocardial reperfusion to observe the effect in mouse myocardial I/R (30mins/120mins) models (Fig. S2A). As shown in Figure 2A, the Ang II level in plasma was significantly higher in I/R group than sham group. Consistent with previous research, I/R model induced significant myocardial infarct size (Fig. 2B-C). The CK-MB level and cTn-I level in plasma were significantly higher in I/R group than sham group (Fig. 2D-E). Echocardiogram showed reduced left ventricular ejection fraction (LVEF), shortened left ventricular fraction (LVFS), and enlarged left ventricular end systolic diameter (LVESd) and left ventricular end diastolic diameter (LVEDd) in I/R group compared with sham group (Fig. 2F-J). Furthermore, myocardial I/R reduced the SOD activity of myocardial tissue, while increased the MDA activity (Fig. 2K-L). In addition, the trends of all these indicators were strengthened by Ang II perfusion, but not by solvent to dissolve Ang II.

### The interaction between CAV-1 protein and ACE2 protein in myocardial I/R injury

**Figure 3:**
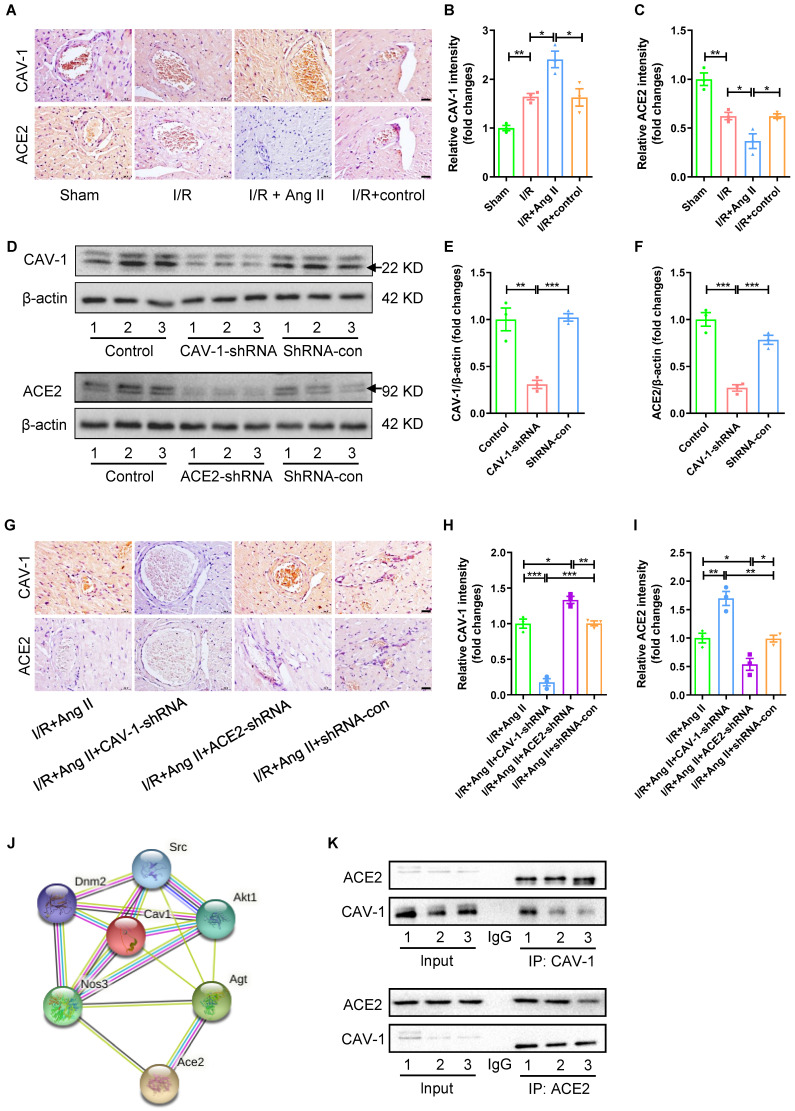
** The proteins interaction between CAV-1 and ACE2 in myocardial I/R injury.** (A-C) Immunohistochemical results of CAV-1 and ACE2 proteins in myocardial tissue, scale bar is 20μm, n = 3. (D-F) The changes in the expression of CAV-1 protein or ACE2 in mouse myocardial tissue after injection of CAV-1-shRNA, ACE2-shRNA, shRNA-con, n = 3. (G-I) Immunohistochemical results of CAV-1 and ACE2 proteins in myocardial tissue, scale bar is 20μm, n = 3. (J) STRING database analysis results of mouse CAV-1 and ACE2 interaction network. (K) The Co-immunoprecipitation experiments of proteins interaction between CAV-1 and ACE2, n = 3. The results are expressed as the mean±SEM. * *p* < 0.05, ** *p* < 0.01, *** *p* < 0.001 by one-way ANOVA (Tukey's test).

Accompanied by I/R-induced myocardial injury, the expression of CAV-1 protein in myocardial tissue was significantly increased, while the expression of ACE2 was significantly reduced. The trends of protein expression were strengthened by Ang II perfusion, but not by solvent to dissolve Ang II (Fig. 3A-C). However, the role of CAV-1 and ACE2 in the aggravation of myocardial I/R injury by Ang II infusion was still unclear. To observe the interaction between CAV-1 and ACE2 in the aggravation of myocardial I/R injury by Ang II infusion, CAV-1-shRNA or ACE2-shRNA were injected into mice to knockdown the mRNA levels and protein expression of CAV-1 or ACE2, and shRNA-con were injected into mice as a viral vector control (Fig. S2B-C, Fig. 3D-F).

In addition, western blot results showed that CAV-1 deficiency promoted the protein expression of ACE2, and knockdown expression of ACE2 also increased protein expression of CAV-1 during myocardial I/R injury under Ang II infusion (Fig. 3G-I). STRING database analysis results of mouse CAV-1 and ACE2 interaction network showed that CAV-1 might interact with ACE2 (Fig. 3J). Co-immunoprecipitation experiments were used to further observe the interaction between CAV-1 protein and ACE2 protein, the results showed that CAV-1 protein pulled down ACE2 protein, and ACE2 protein also pulled down CAV-1 protein (Fig. 3K).

### Knockdown of CAV-1 reduces, while ACE2 silence strengthens the myocardial I/R injury aggravated by Ang II

**Figure 4:**
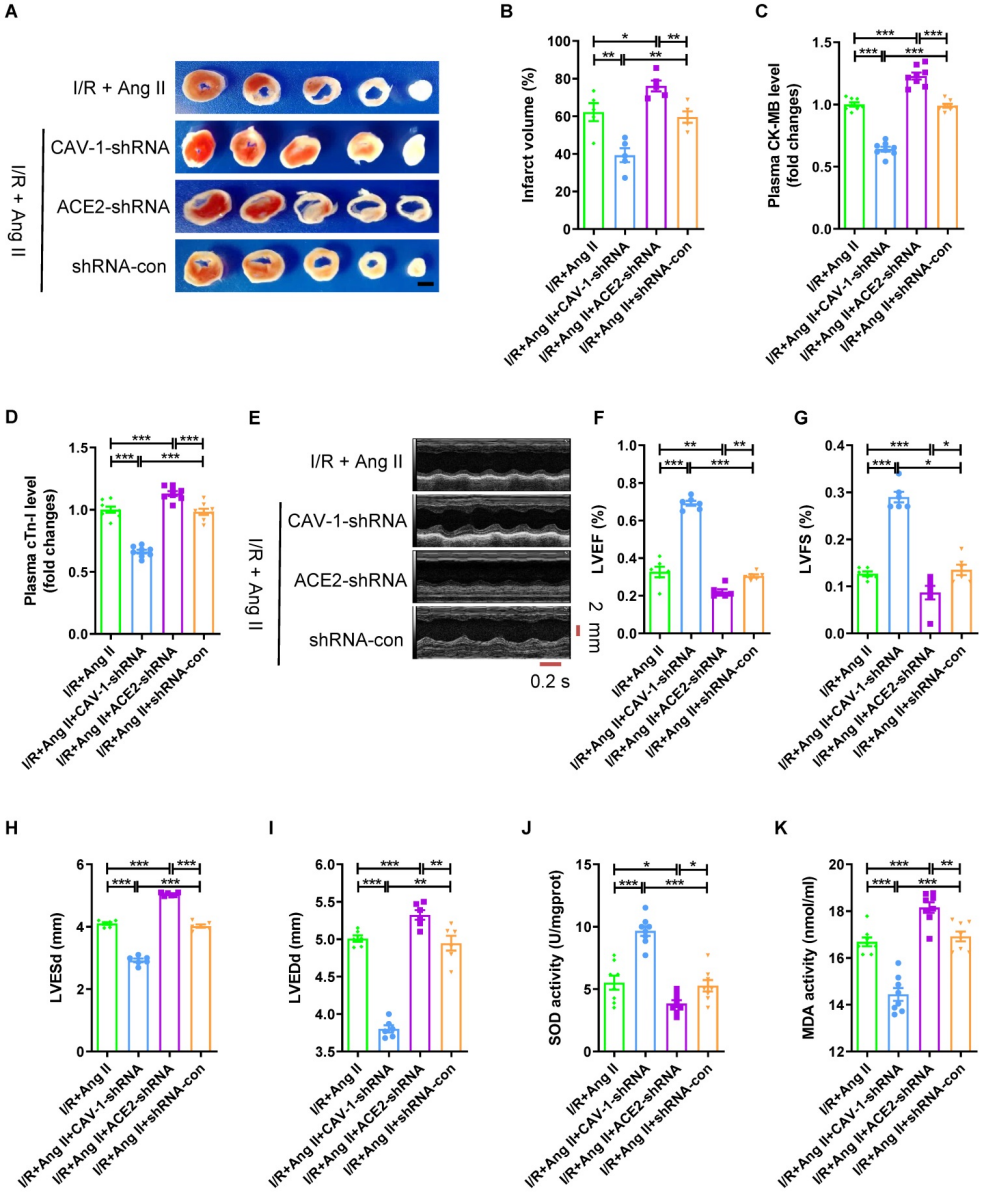
** Knockdown of CAV-1 reduces, while ACE2 silence strengthens the myocardial I/R injury aggravated by Ang II.** (A-B) TTC staining and myocardial infarction volume of mouse myocardial tissue, scale bar is 5 mm, n = 5. (C-D) CK-MB levels and cTn-I levels in mouse plasma. (E-I) Cardiac function indicators left ventricular ejection fraction (LVEF), left ventricular fraction (LVFS), left ventricular end systolic diameter (LVESd) and left ventricular end diastolic diameter (LVEDd) were assessed by echocardiography, time stamp is 0.2 s and scale bar is 2 mm. (J-K) The superoxide dismutase (SOD) activity and malondialdehyde (MDA) activity of myocardial tissue. The results are expressed as the mean±SEM, n = 8. * *p* < 0.05, ** *p* < 0.01, *** *p* < 0.001 by one-way ANOVA (Tukey's test).

Then we observed the role of CAV-1/ACE2 axis played in the aggravation of myocardial I/R injury by Ang II infusion (Fig. S2D). Compared to I/R + Ang II group, CAV-1-shRNA decreased myocardial infarct size (Fig. 4A-B), reduced the plasma CK-MB level and cTn-I level (Fig. 4C-D), increased LVEF and LVFS, and decreased LVESd and LVEDd (Fig. 4E-I); while ACE2-shRNA increased myocardial infarct size (Fig. 4A-B), raised the plasma CK-MB level and cTn-I level (Fig. 4C-D), decreased LVEF and LVFS, and enlarged LVESd and LVEDd (Fig. 4E-I). Furthermore, CAV-1-shRNA increased the SOD activity and reduced MDA activity of myocardial tissue, while ACE2-shRNA decreased the SOD activity and increased MDA activity (Fig. 4J-K). These demonstrated that CAV-1 deficiency reduced, but knockdown expression of ACE2 aggravated myocardial I/R injury under Ang II infusion.

### C3 reverses elevated Ang II levels and myocardial I/R injury

**Figure 5:**
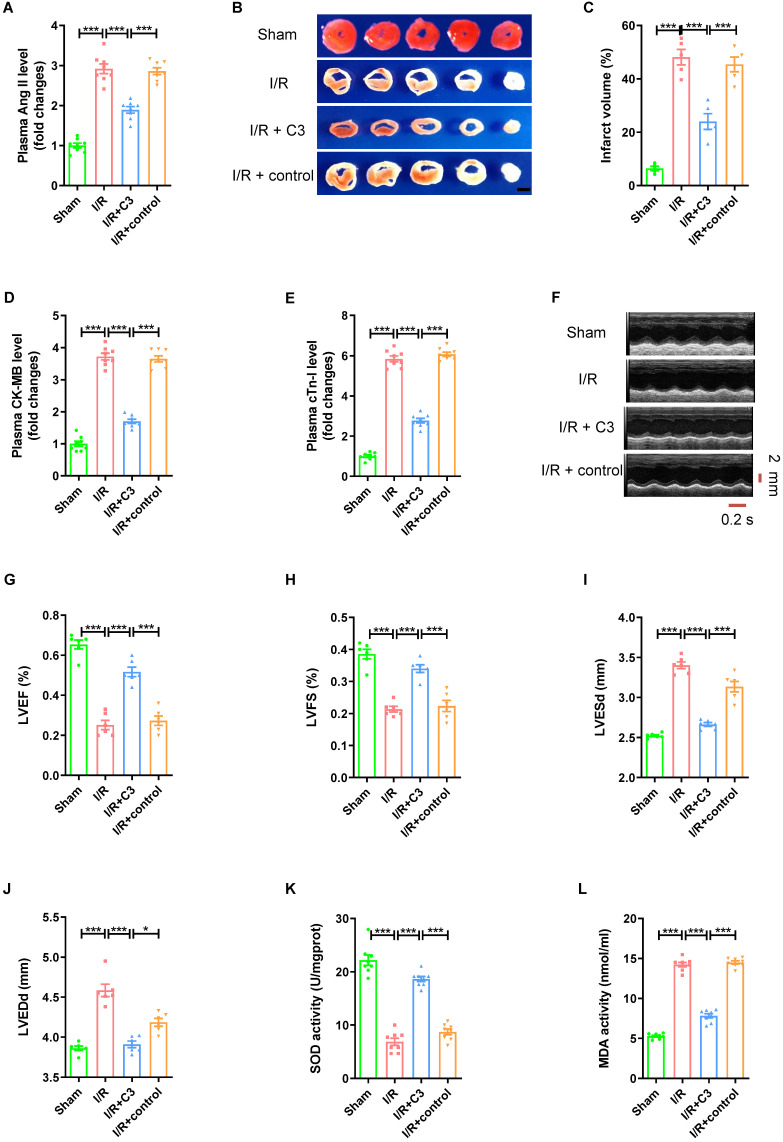
** C3 reverses elevated Ang II levels and myocardial I/R injury.** (A) Ang II levels in mouse plasma. (B-C) TTC staining and myocardial infarction volume of mouse myocardial tissue, scale bar is 5 mm, n = 5. (D-E) CK-MB levels and cTn-I levels in mouse plasma. (F-J) Cardiac function indicators left ventricular ejection fraction (LVEF), left ventricular fraction (LVFS), left ventricular end systolic diameter (LVESd) and left ventricular end diastolic diameter (LVEDd) were assessed by echocardiography, time stamp is 0.2 s and scale bar is 2 mm. (K-L) The superoxide dismutase (SOD) activity and malondialdehyde (MDA) activity of myocardial tissue. The results are expressed as the mean±SEM, n = 8. * *p* < 0.05, ** *p* < 0.01, *** *p* < 0.001 by one-way ANOVA (Tukey's test).

As a metabolite of intestinal flora, C3 has been proven to be involved in regulating blood pressure. Mice that drank sodium propionate aqueous solution for 15 days (Fig. S3A) had significantly lower plasma Ang II level (Fig. 5A), less myocardial infarct size (Fig. 5B-C), lower plasma CK-MB level and cTn-I level (Fig. 5D-E), higher LVEF and LVFS, and smaller LVESd and LVEDd (Fig. 5F-J) after establishing an myocardial I/R model than mice that drank sterile water or mice that drank sodium chloride aqueous solution. Furthermore, compared to I/R group or I/R + control group, C3 increased the SOD activity, and reduced MDA activity of myocardial tissue (Fig. 5K-L).

### The C3 content in preoperative stool of patients with CPB is correlated with the degree of myocardial I/R injury

**Figure 6:**
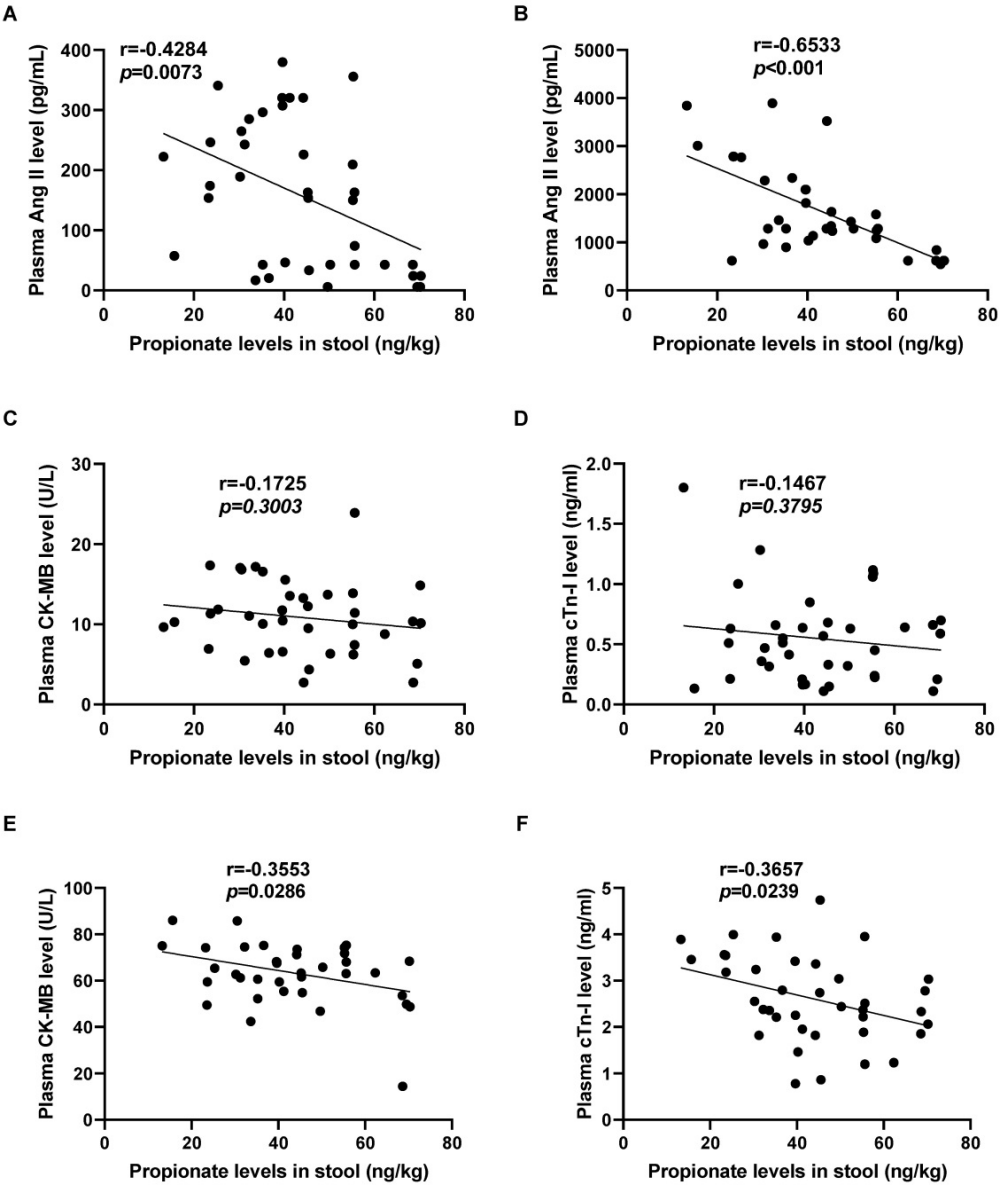
** The C3 content in preoperative stool of patients with CPB is correlated with the degree of myocardial I/R injury.** (A-B) The correlation analysis between the level of propionate in the patient's feces before surgery and the level of Ang II in the patient's plasma before surgery (T0, A), and at the time of myocardial reperfusion (T3, B). (C-F) The correlation analysis between the level of propionate in the patient's feces before surgery and the level of CK-MB, cTn-I in the patient's plasma before surgery (T0, C&D), and at the time of myocardial reperfusion (T3, E&F). The results are expressed as the mean ± SEM, n = 38. * *p* < .05, ** *p* < .01, *** *p* < .001 by spearman analysis.

To further observe the relationship between propionate content and myocardial I/R injury, we analyzed the correlation between the level of C3 in the feces of CPB patients before the operation and the level of myocardial I/R injury markers in the plasma at T0 or at T3. The C3 content in the patient's feces before surgery is significantly negatively correlated with the Ang II level in the plasma at T0 (r=-0.4284, *p*=0.0073; Fig. 6A) and at T3 (r=-0.6533, *p*<0.001; Fig. 6B). Furthermore, there is no significant correlation between the C3 content in the patient's feces before surgery and CK-MB level (r=-0.1725, *p*=0.3003; Fig. 6C), cTn-I level (r=-0.1467, *p*=0.3795; Fig. 6D) in the patient's plasma before the operation at T0. While the C3 content in preoperative stool were negatively correlated with plasma CK-MB level (r=-0.3553, *p*=0.0286; Fig. 6E) and plasma cTn-I level (r=-0.3657, *p*=0.0239; Fig. 6F) at T3.

### C3 reduces Ang II levels and myocardial I/R injury through GPR41

**Figure 7:**
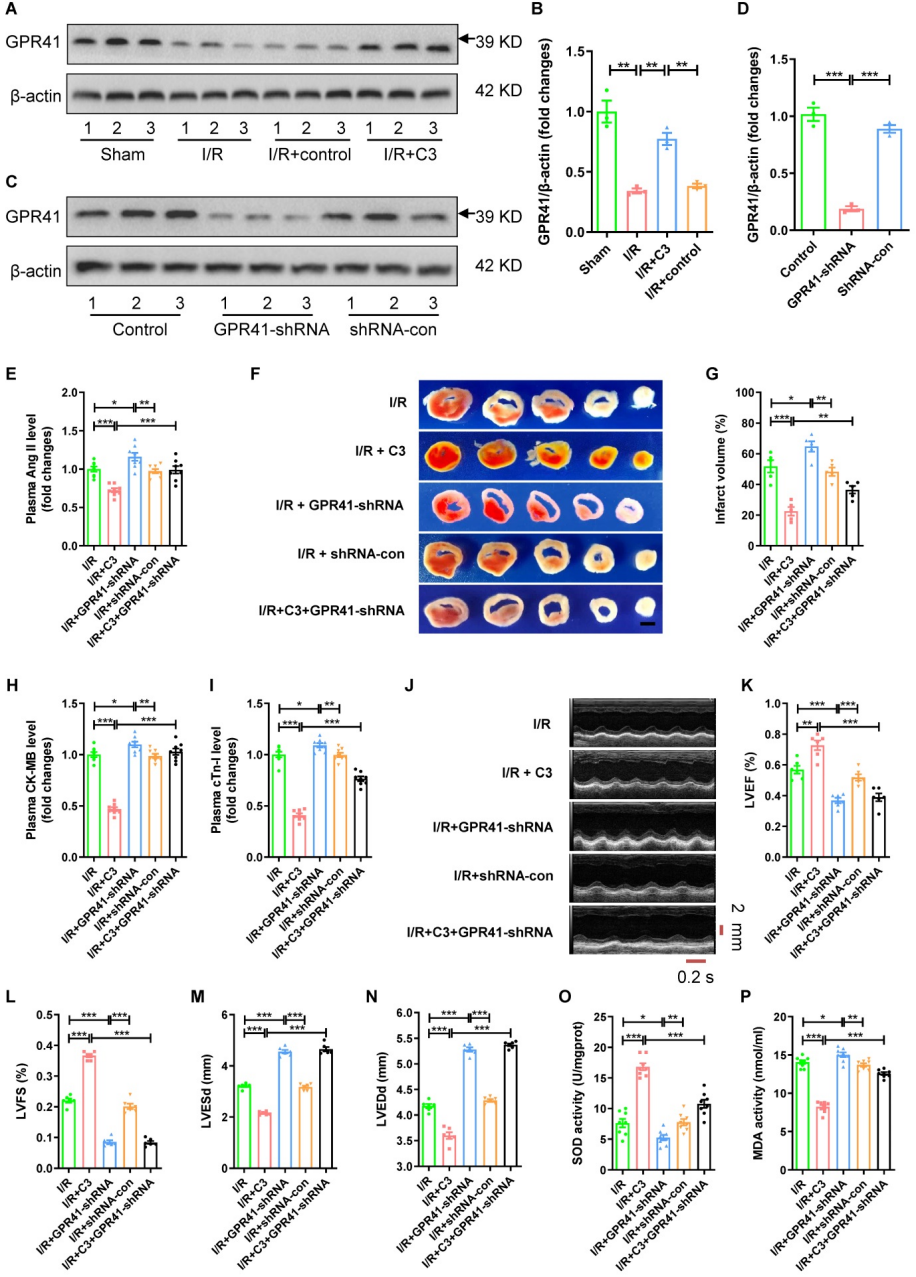
**C3 reduces Ang II levels and myocardial I/R injury through GPR41.** (A-B) Changes in GPR41 expression in mouse myocardial tissue after C3 treatment during myocardial I/R; n = 3. (C-D) The changes in the expression of GPR41 in mouse myocardial tissue after injection of GPR41-shRNA, shRNA-con; n = 3. (E) Ang II levels in mouse plasma. (F-G) TTC staining and myocardial infarction volume of mouse myocardial tissue, scale bar is 5 mm, n = 5. (H-I) CK-MB levels and cTn-I levels in mouse plasma. (J-N) Cardiac function indicators left ventricular ejection fraction (LVEF), left ventricular fraction (LVFS), left ventricular end systolic diameter (LVESd) and left ventricular end diastolic diameter (LVEDd) were assessed by echocardiography, time stamp is 0.2 s and scale bar is 2 mm. (O-P) The superoxide dismutase (SOD) activity and malondialdehyde (MDA) activity of myocardial tissue. The results are expressed as the mean±SEM, n = 8. * *p* < 0.05, ** *p* < 0.01, *** *p* < 0.001 by one-way ANOVA (Tukey's test).

Compared to I/R group or I/R + control group, C3 increased the protein expression of GPR41 in myocardial tissue (Fig. 7A-B). GPR41-shRNA or shRNA-con were injected into myocardial tissue to observe the role of GPR41 in the protective effect of C3 on myocardial I/R injury (Fig. 7C-D; Fig. S3B-C). Compared to I/R group, C3 decreased plasma Ang II level (Fig. 7E), reduced myocardial infarct size (Fig. 7F-G) and plasma CK-MB level and cTn-I level (Fig. 7H-I), increased LVEF and LVFS, reduced LVESd and LVEDd (Fig. 7J-N), increased the SOD activity, and reduced MDA activity of myocardial tissue (Fig. 7O-P); while GPR41-shRNA increased plasma Ang II level (Fig. 7E), increased myocardial infarct size (Fig. 7F-G) and plasma CK-MB level and cTn-I level (Fig. 7H-I), decreased LVEF and LVFS, increased LVESd and LVEDd (Fig. 7J-N), decreased the SOD activity, and increased MDA activity of myocardial tissue (Fig. 7O-P). Furthermore, plasma Ang II level was lower (Fig. 7A), myocardial infarct size (Fig. 7F-G) was smaller and plasma CK-MB level and cTn-I level (Fig. 7H-I) were lower, LVEF and LVFS were higher, and LVESd and LVEDd were shorter (Fig. 7J-N), the SOD activity of myocardial tissue was higher, and the MDA activity of myocardial tissue was lower in I/R + C3 group than that in I/R + C3 + GPR41-shRNA group (Fig. 7O-P). Consistent with GPR41-shRNA significantly abolished the protective effect of C3 on myocardial I/R injury, GPR41-shRNA significantly reversed the decrease in CAV-1 expression and the increase in ACE2 expression during myocardial I/R caused by C3 treatment (Fig. S4A-C).

## Discussion

In this study, we found that elevated Ang II aggravated myocardial reperfusion injury in mice, and the level of Ang II was positively correlated with the level of myocardial injury during CPB patient's reperfusion. Furthermore, CAV-1 deletion alleviated, while ACE2 deletion enhanced Ang II-induced myocardial I/R injury in mice, and the interaction experiment between proteins showed that there is a direct interaction between CAV-1 and ACE2, these indicate that CAV-1/ACE2 axis plays an important regulatory role in myocardial I/R injury aggravated by Ang II. In addition, we uncovered that C3 inhibited the increase level of Ang II through GPR41 during myocardial reperfusion, thereby reducing myocardial I/R injury in mice. Clinically, the level of C3 in preoperative stool was negatively correlated with the level of Ang II and myocardial I/R injury markers in plasma during CPB patient's reperfusion. Therefore, this study reveals that reasonable dietary control methods, such as dietary fiber or sodium propionate aqueous solution, to promote the increase of C3 levels are potential strategies for the treatment of myocardial I/R injury. And we confirmed the important role of C3 in regulating Ang II and the important mechanism of Ang II aggravating myocardial injury during myocardial I/R.

Ang II is the most important component of angiotensin, and it is also one of the strongest vasoconstrictor substances known. Consistent with our findings, some studies have also suggested the role of Ang II in myocardium I/R injury. Li et al. revealed that losartan, an angiotensin II (Ang II) receptor blocker acting on the Ang II type-1 receptor (AT1R) subtype, protects against myocardial I/R injury via vascular integrity preservation 42. Liu et al. showed that inhibiting Ang II can improve ventricular remodeling in murine models of myocardial I/R injury 43. These indicate that maintaining a steady state of Ang II levels is a potential strategy for treating myocardial I/R injury. However, the underlying mechanism of Ang II's role in cardiovascular injury during myocardial I/R has not yet been elucidated. CAV-1, as an important membrane protein for cell membrane signal transduction, can adsorb and bind eNOS protein to reduce the level of NO in plasma, while NO as an important vasodilator in plasma can counteract the vasoconstrictive effect of Ang II. Meanwhile, ACE2 can hydrolyze Ang II to Ang (1-7), and then act on the Mas receptor to relax the blood vessels, anti-proliferative and anti-oxidative stress 13, 44. Not only CAV-1, CAV-3 has also been confirmed to play an important role in myocardial I/R injury. It has been found that promoting the expression of CAV-3 significantly reduces myocardial dysfunction and I/R injury 45, hyperglycemia-induced oxidative stress and I/R injury 46, suppresses autophagy and death of cardiomyocytes 47. Markandeya et al. found that stable CAV-3 expression is essential for protecting the signaling mechanisms in pharmacologically and pressure overload-induced cardiac hypertrophy 48. CAV-1 is mainly expressed in endothelial cells, adipocytes, fibroblasts, etc., while the expression of CAV-3 has muscle cell specificity (cardiomyocytes, smooth muscle cells, etc.). In this study, we have not only confirmed the regulatory role of CAV-1 and ACE2 in Ang II aggravated myocardial I/R injury, but also confirmed the interaction between CAV-1 and ACE2 protein, and clarified a new mechanism that Ang II aggravates myocardial I/R injury, providing a potential target for treating myocardial I/R injury. Meanwhile, some studies have found that inhibition of Ang II production increases susceptibility to acute ischemia/reperfusion arrhythmias 49. Therefore, regulating Ang II at a relatively stable level is essential for the prevention and treatment of myocardial I/R injury.

To a large extent, our health also depends on the gut flora. This is because the gut flora helps the body use food and produce the necessary micronutrients, including vitamins. Gut bacteria can produce many easily metabolizable substances from dietary fiber, including C3. These materials have energy storage effects, can reduce intestinal osmotic pressure, and play an important role in regulating colorectal function and colonic epithelial cell shape. With the in-depth study of intestinal flora and its metabolites, more and more studies have confirmed that metabolites of intestinal flora can regulate cardiovascular injury. C3-fed mice had reduced cardiac hypertrophy, fibrosis, vascular dysfunction, and hypertension in the hypertensive model compared to control mice, which also reduced susceptibility to arrhythmias and atherosclerosis lesions.^39^ In addition, C3 affects the release of renin and the level of Ang II in plasma through the kidney olfr78 receptor.^36^ In this study, we show that C3 inhibits the rapid increase of Ang II levels during reperfusion and reduces myocardial I/R injury through GPR41. In addition to GPR41, some other G protein-coupled receptors have also been found to play an important role in the regulation of blood pressure or cardiovascular disease by SCFA. The adverse effects of a low-fiber Westernized diet may lead to high blood pressure through SCFA production and insufficient GPR43/109A signaling, suggesting that maintaining a healthy SCFA-producing microbiota is important for cardiovascular health 50. Acetate and butyrate improve endothelial dysfunction induced by AngII by increasing the bioavailability of NO. The effect of butyrate seems to be related to GPR41/43 activation, whereas acetate effects were independent of GPR41/43 34. This suggests that different SCFAs may have different mechanisms to regulate blood pressure or Ang II levels. In daily life, dietary fiber is a good source of C3. Whole grain foods and fruits contain cellulose and inulin fiber, and the gut flora produces a short-chain fatty acid during the digestion of these ingredients, which is the C3 in the study 51, 52. Meanwhile, lecithin, choline, and carnitine contained in foods can eventually be transformed into trimethylamine N-oxide (TMAO) through metabolism of the intestinal flora and liver transformation, which has been proven to participate in promoting a variety of cardiovascular disease progression 53-55. All these indicate that differences in diet may lead to different metabolites of the flora, which in turn affects the outcome of cardiovascular disease. This study opens up a new way to treat myocardial I/R disease, indicates the importance of reasonable diet, maintaining the homeostasis of intestinal flora and promoting the production of beneficial metabolites for the treatment of patients with myocardial I/R injury.

This study also has some limitations. In this study, we only observed the role of C3 in myocardial I/R, but it is not clear whether other components in SCFA also have similar effects to C3. This study did not reveal how GPR41 regulates Ang II levels in plasma and the protein interaction between GPR41 and CAV-1 or ACE2. We focused on the role of GPR41 in the regulation of Ang II levels in myocardial I/R injury by propionic acid, but we did not observe whether GPR43 or GPR109A also has a similar effect. Therefore, the role of GPR43 or GPR109A in reducing myocardial I/R injury by propionate has not yet been determined. In addition, in order to avoid the interference of estrogen on the experiment, this study only used male mice as the research object. CAV-3 and CAV-1 are both caveolins played important roles in myocardial I/R injury, but this study did not involve the expression changes and underlying mechanisms of CAV-3. The clinical transformation and application of intestinal flora and its metabolites is a direction worth exploring in the future.

In summary, we revealed that Ang II relied on the CAV-1/ACE2 axis to aggravate myocardial I/R injury, elucidating a new mechanism of myocardial I/R injury; confirmed that C3 regulates Ang II levels and myocardial I/R injury through GPR41, providing a new direction and basis for the prevention and treatment of myocardial I/R injury.

## Supplementary Material

Supplementary figures.
