# Supplementary material for: Propionate alleviates myocardial ischemia-reperfusion injury aggravated by Angiotensin II dependent on caveolin-1/ACE2 axis through GPR41
Source: Int J Biol Sci. 2022 Jan 1;18(2):858–72. doi: 10.7150/ijbs.67724 (PMC8741842; doi:10.7150/ijbs.67724)
Supplement: Supplementary file 1 — Supplementary figures. [file ijbsv18p0858s1.pdf]

Supplementary Fig. legend

Supplementary Fig. S1: Heart rate and blood pressure of patients undergoing cardiopulmonary bypass. (A) Heart rate of patients. (B-D) Systolic blood pressure, diastolic blood pressure and mean arterial pressure of patients. The results are expressed as the mean±SEM, n = 38. * $p < 0.05$, ** $p < 0.01$, *** $p < 0.001$.

Supplementary Fig. S2: CAV-1/ACE2 axis plays an important regulatory role in myocardial ischemia/reperfusion (I/R) injury aggravated by Ang II perfusion. (A) The experimental design of observing the role of Ang II perfusion played in the myocardial I/R injury in mice. (B-C) The relative mRNA levels of ACE2 and CAV-1. (D) The experimental design of observing the role of CAV-1 and ACE2 in the aggravation of myocardial I/R injury by Ang II perfusion. The results are expressed as the mean±SEM, n = 8. * $p < 0.05$, ** $p < 0.01$, *** $p < 0.001$.

Supplementary Fig. S3: Propionate (C3) inhibited the increase level of Ang II through GPR41 during myocardial reperfusion (A) The experimental design of observing the effect of C3 on myocardial I/R injury. (B) The experimental design of observing the role of GPR41 in C3 reducing Ang II levels and myocardial I/R injury. (C) The relative mRNA levels of ACE2 and CAV-1. The results are expressed as the mean±SEM, n = 8. * $p < 0.05$, ** $p < 0.01$, *** $p < 0.001$.

Supplementary Fig. S4: The effect of C3 and GPR41 on the protein expression

levels of CAV1 and ACE in myocardial tissue during myocardial I/R. (A-C) CAV-1 and ACE2 immunohistochemical staining and intensity quantification analysis of myocardial tissue in mice with I/R group, I/R + C3 group, I/R + GPR41-shRNA group, I/R + shRNA-con group, I/R+ C3 + GPR41-shRNA. scale bar is 20 μm (n = 3). The results are expressed as the mean±SEM. * $p < 0.05$, ** $p < 0.01$, *** $p < 0.001$.

Table S1 Patient characteristics

Parameter	
Females (n)	17
Males (n)	21
Ages, yr	50 (36-61)
Body mass index, kg m^{-2}	23.90 (18.66-28.73)
Duration of hospitalization, days	22.25 (17-36)
Duration of anesthesia, min	332.44 (274-458)
Duration of surgery, min	272 (221-414)
Duration of CPB, min	127.2 (70-180)
Estimated blood loss, ml	355.5 (300-400)
Duration of ICU, hours	78.57 (39.3-93.33)

Note. Numbers are counts (percentage) or median (interquartile range).

Figure S1

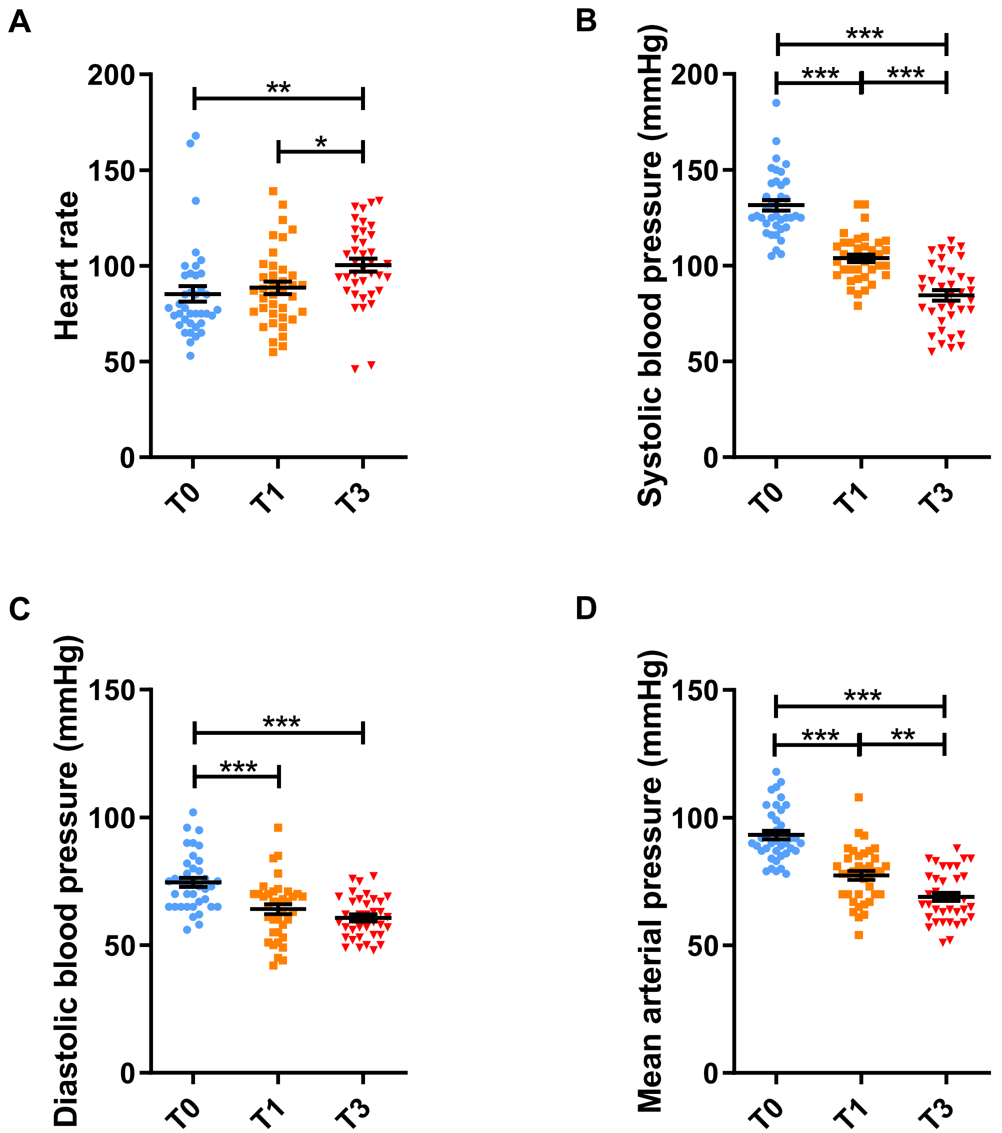

A
Heart rate
200
150
100
50
0
**
*
T0
T1
T3
B
Systolic blood pressure (mmHg)
200
150
100
50
0
***
***
***
T0
T1
T3
C
Diastolic blood pressure (mmHg)
150
100
50
0
***
***
T0
T1
T3
D
Mean arterial pressure (mmHg)
150
100
50
0
***
***
**
T0
T1
T3

Figure S2

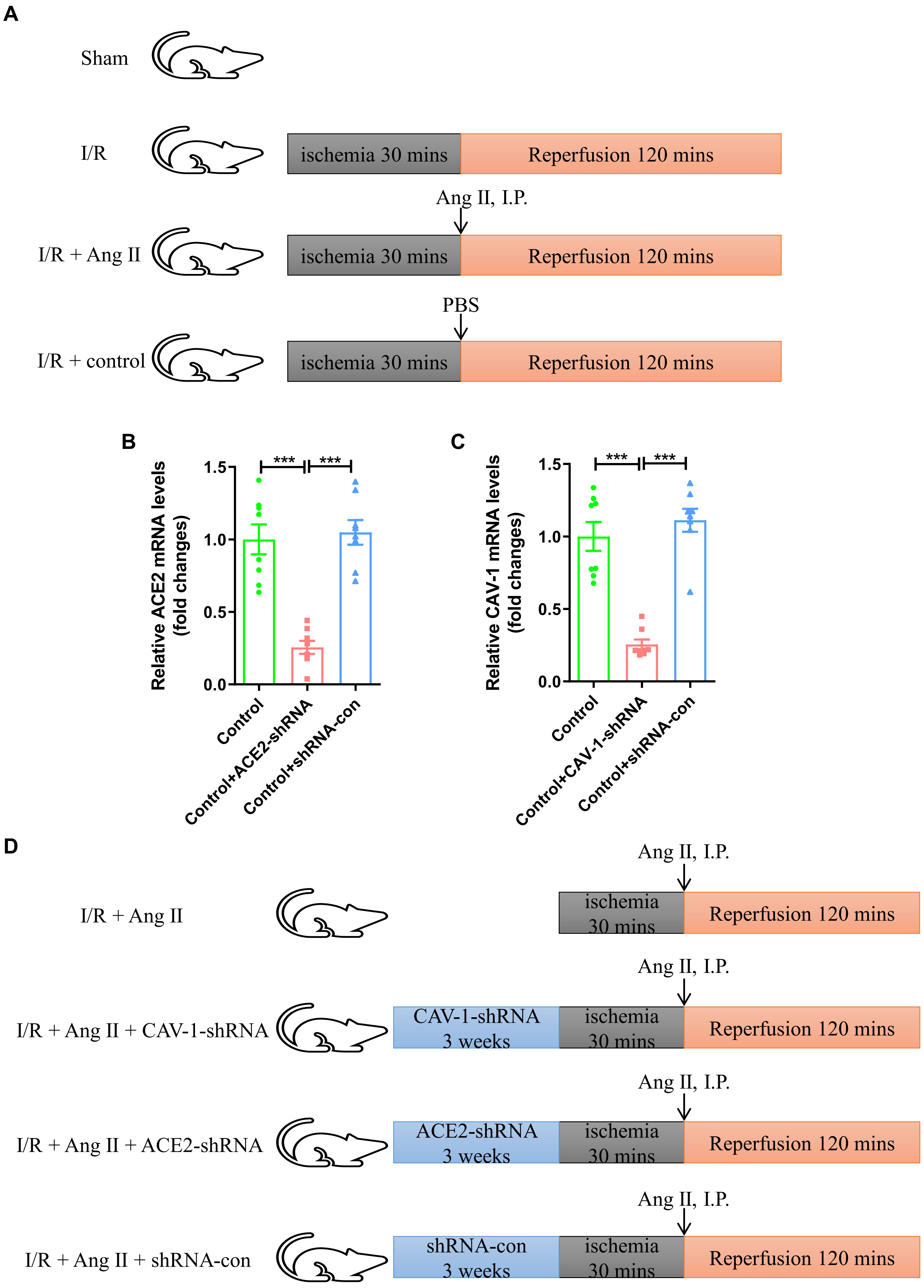
A
Sham
I/R
ischemia 30 mins
Reperfusion 120 mins
Ang II, I.P.
I/R + Ang II
ischemia 30 mins
Reperfusion 120 mins
PBS
I/R + control
ischemia 30 mins
Reperfusion 120 mins
B
Relative ACE2 mRNA levels (fold changes)
1.5
1.0
0.5
0.0
***
***
Control
Control+ACE2-shRNA
Control+shRNA-con
C
Relative CAV-1 mRNA levels (fold changes)
1.5
1.0
0.5
0.0
***
***
Control
Control+CAV-1-shRNA
Control+shRNA-con
D
Ang II, I.P.
I/R + Ang II
ischemia 30 mins
Reperfusion 120 mins
Ang II, I.P.
I/R + Ang II + CAV-1-shRNA
CAV-1-shRNA 3 weeks
ischemia 30 mins
Reperfusion 120 mins
Ang II, I.P.
I/R + Ang II + ACE2-shRNA
ACE2-shRNA 3 weeks
ischemia 30 mins
Reperfusion 120 mins
Ang II, I.P.
I/R + Ang II + shRNA-con
shRNA-con 3 weeks
ischemia 30 mins
Reperfusion 120 mins

Figure S3

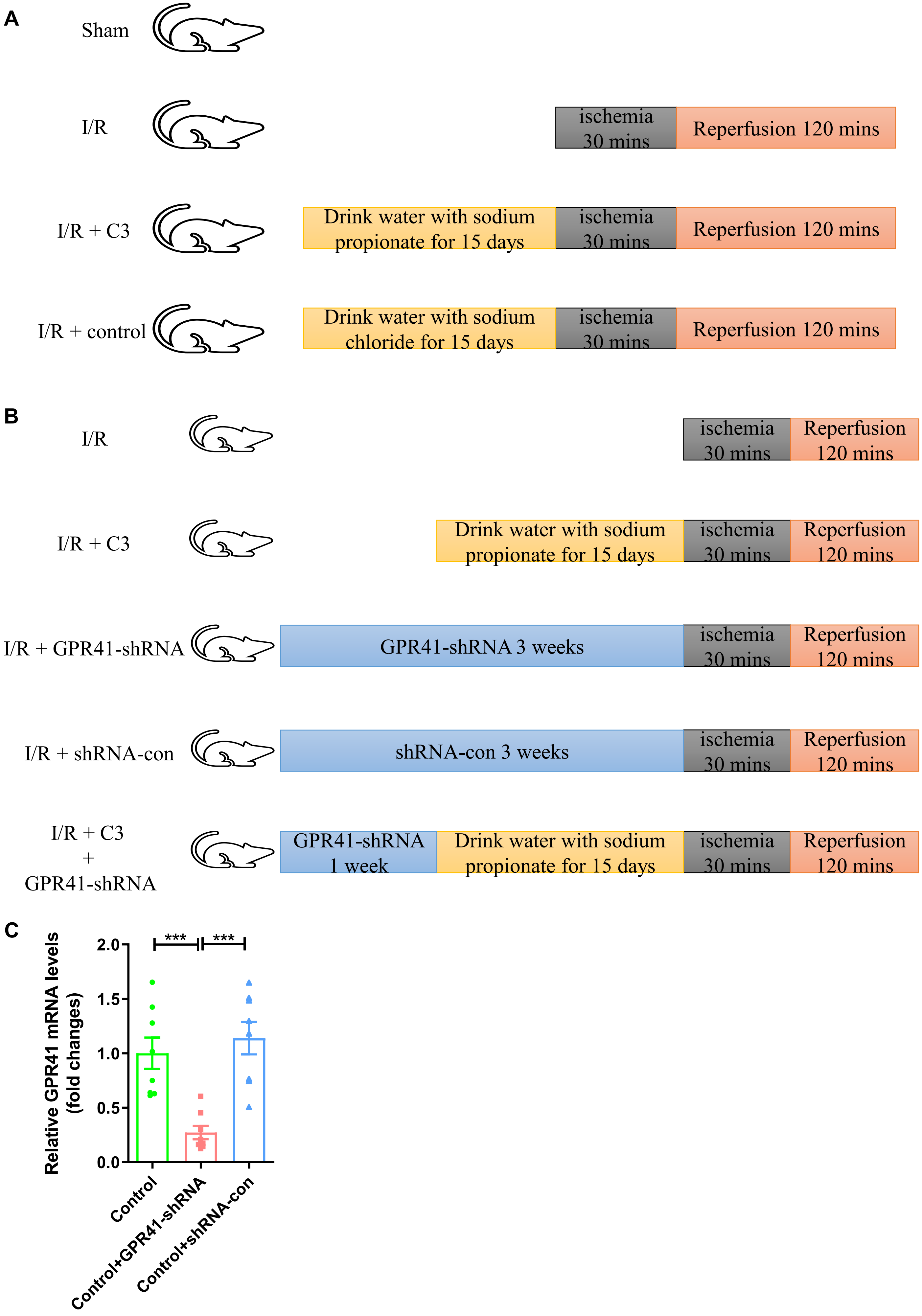

A
Sham
I/R
ischemia 30 mins
Reperfusion 120 mins
I/R + C3
Drink water with sodium propionate for 15 days
ischemia 30 mins
Reperfusion 120 mins
I/R + control
Drink water with sodium chloride for 15 days
ischemia 30 mins
Reperfusion 120 mins
B
I/R
ischemia 30 mins
Reperfusion 120 mins
I/R + C3
Drink water with sodium propionate for 15 days
ischemia 30 mins
Reperfusion 120 mins
I/R + GPR41-shRNA
GPR41-shRNA 3 weeks
ischemia 30 mins
Reperfusion 120 mins
I/R + shRNA-con
shRNA-con 3 weeks
ischemia 30 mins
Reperfusion 120 mins
I/R + C3 + GPR41-shRNA
GPR41-shRNA 1 week
Drink water with sodium propionate for 15 days
ischemia 30 mins
Reperfusion 120 mins
C
Relative GPR41 mRNA levels (fold changes)
2.0
1.5
1.0
0.5
0.0
***
***
Control
Control+GPR41-shRNA
Control+shRNA-con

Figure S4

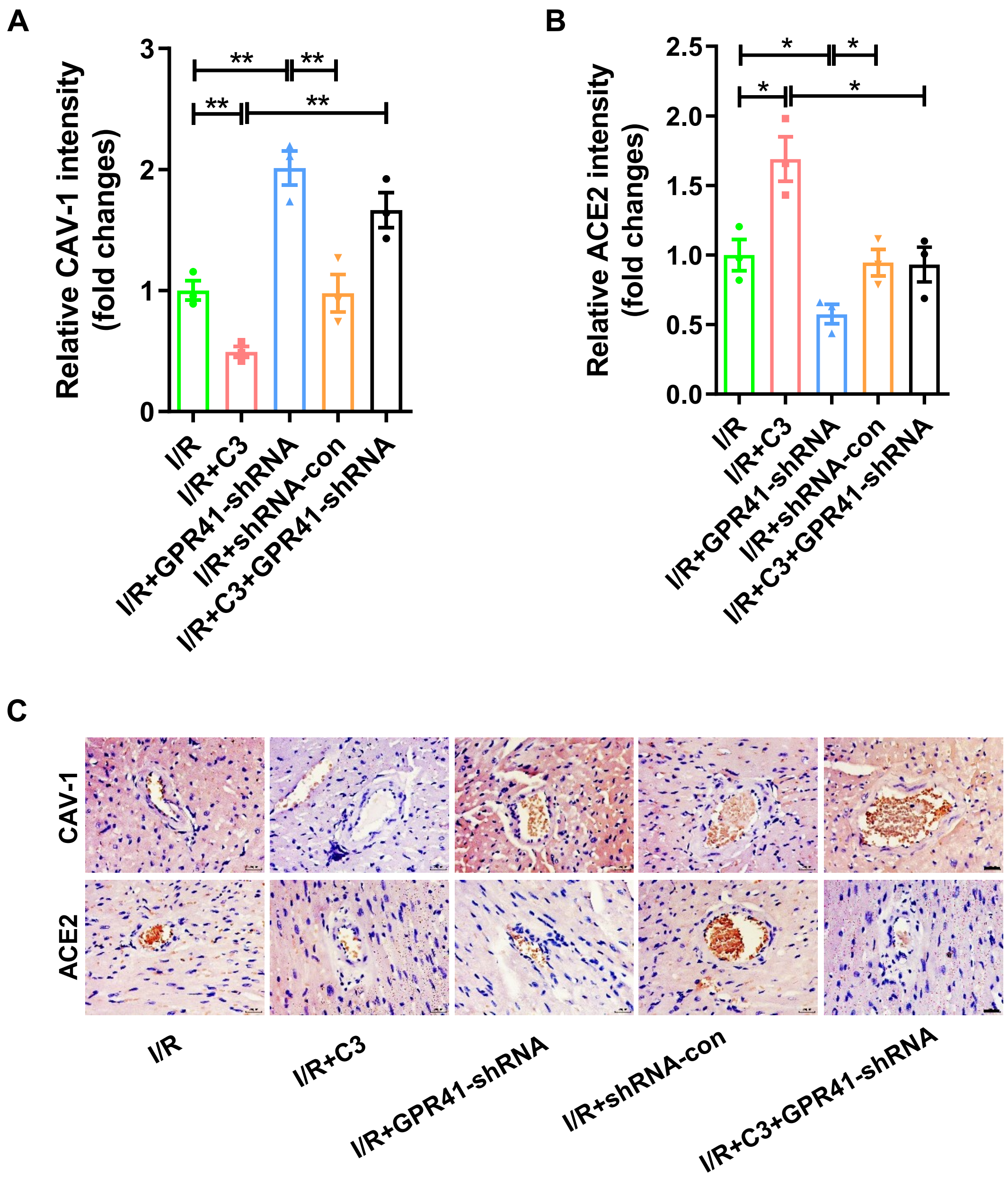

A
Relative CAV-1 intensity (fold changes)
0
1
2
3
**
**
**
**
I/R
I/R+C3
I/R+GPR41-shRNA
I/R+shRNA-con
I/R+C3+GPR41-shRNA
B
Relative ACE2 intensity (fold changes)
0.0
0.5
1.0
1.5
2.0
2.5
*
*
*
*
I/R
I/R+C3
I/R+GPR41-shRNA
I/R+shRNA-con
I/R+C3+GPR41-shRNA
C
CAV-1
ACE2
I/R
I/R+C3
I/R+GPR41-shRNA
I/R+shRNA-con
I/R+C3+GPR41-shRNA
